# Changes in life satisfaction during the transition to retirement: findings from the FIREA cohort study

**DOI:** 10.1007/s10433-022-00745-8

**Published:** 2022-11-23

**Authors:** K. C. Prakash, Marianna Virtanen, Soili Törmälehto, Saana Myllyntausta, Jaana Pentti, Jussi Vahtera, Sari Stenholm

**Affiliations:** 1 Unit of Health Sciences, Faculty of Social Sciences, Tampere University, C-319, Arvo Ylpönkatu 34, 33520 Tampere, Finland; 2 Gerontology Research Center, Tampere University, Tampere, Finland; 3 Department of Public Health, University of Turku and Turku University Hospital, Turku, Finland; 4 Department of Psychology, Stress Research Institute, Stockholm University, Stockholm, Sweden; 5 School of Educational Sciences and Psychology, University of Eastern Finland, Joensuu, Finland; 6 Division of Insurance Medicine, Karolinska Institutet, Stockholm, Sweden; 7 Department of Psychology and Speech-Language Pathology, University of Turku, Turku, Finland; 8 Centre for Population Health Research, University of Turku and Turku University Hospital, Turku, Finland; 9 Clinicum, Faculty of Medicine, University of Helsinki, Helsinki, Finland

**Keywords:** Life-changes, Well-being, Longitudinal studies, Spousal-status, Self-rated health

## Abstract

Life satisfaction is an essential construct of well-being that is tied to behavioral, emotional, social and psychological outcomes. This study aimed to examine changes in total and domain-specific life satisfaction during the retirement transition and additionally examine whether those changes differ by gender, occupation, health and spousal working status. Aging public sector employees (*n* = 3543) from the Finnish Retirement and Aging Study cohort study were followed up annually before and after retirement. Total life satisfaction score (range 4–20) was computed by summing up the responses in four domains (interestingness, happiness, easiness and togetherness). The mean and mean change estimates and their 95% CI were calculated by using the linear regression models with generalized estimating equations, adjusted for age, gender, occupation, health and marital status. Total life satisfaction score improved among the entire study population during the retirement transition and remained stable thereafter. The improvement was greater among women versus men (gender * time interaction *p* = 0.004), among those with suboptimal health before retirement vs. those who had good (health * time *p* < 0.0001) and those who had no spouse vs. those who had a retired or working spouse (spousal-status * time *p* < 0.0001). In case of domain-specific life satisfaction scores, the greatest improvement was observed in the easiness domain. Life satisfaction improves during the retirement transition period, especially among women, those with suboptimal health and those living without a spouse. The improvement was considerably greater in the easiness domain than any other domains. Life satisfaction remained improved and stable during the post-retirement period.

**Supplementary Information:**

The online version contains supplementary material available at 10.1007/s10433-022-00745-8.

## Introduction

Retirement is a major life transition during the late mid-life and can have both beneficial and adverse health effects (van der Heide et al. 2013; Stenholm et al. 2020). In addition to different aspects of health, life satisfaction is an essential construct in positive psychology (Gilman and Huebner 2003) and should be considered in the context of retirement transition. Life satisfaction is a key indicator of well-being that is tied to behavioral, emotional, social and psychological outcomes (Proctor et al. 2008). Life satisfaction emphasizes an individual’s ability to cope against the development of psychopathological problems and is assessed through anticipated subjective feeling (Moen 1996; Pavot and Diener 2008; Proctor et al. 2008). Generally, happiness and achievement of good life are linked to the positive evaluations of life satisfaction, and on the contrary, unhappiness increases the tendency of negative evaluations of life satisfaction. These findings suggest that life satisfaction is a subjective evaluation of quality of life and a key component of subjective well-being (Diener and Diener, 1995). In fact, the components of subjective well-being are often synonymous to happiness and mostly used as substitutes of each other (Seligman 2002).

Previous studies have examined changes in life satisfaction during the retirement transition (Calasanti et al. 2021; Carr et al. 2020; Gorry et al. 2018; Hansson et al. 2018; Hershey and Henkens 2014; Pinquart and Schindler 2007; Weber and Hülür 2020) showing that life satisfaction improves during the transition period. However, the previous studies have relied on a single item to measure life satisfaction. The only exception is the study by Calasanti et al. (2021), where they studied the change in domain-specific life satisfaction (leisure, family and finance) during retirement transition using longitudinal sample drawn from the US Health and Retirement Study (HRS). The study reported higher improvement in life satisfaction among those who were satisfied with their financial status before retirement. This implies that the measurement of life satisfaction is a sensitive approach, and several domains of life could influence the change in life satisfaction during retirement transition. Therefore, additional use of domain-specific measures of life satisfaction is equally important.

Moreover, the changes in life satisfaction during retirement transition in previous studies have been attributed to variations in resources and characteristics such as gender, health and marital status. According to the theory of perspective on retirement, gender and well-being, by Moen (1996), men and women who retire from their career jobs are vulnerable to feelings of role loss, which can lead to decreased mental well-being of an individual (Moen 1996). The transition to retirement can come with a different feeling for women compared to men (Kim and Moen 2002; Price and Joo 2005) as women may come to retirement with lower level of personal control than men (Kim and Moen 2002). However, the process of retirement may represent reduction in role strain and gain in time resources in case of both genders. Previous studies have reported gender difference in changes in well-being during retirement transition (Butterworth et al. 2006; Calasanti et al. 2021; Coursolle et al. 2010; Kim and Moen 2002; Potočnik et al. 2013). The improvement in life satisfaction during the retirement transition (Calasanti et al. 2021), emotional well-being (Coursolle et al. 2010) and psychological well-being (Kim and Moen 2002) has been reported higher among men compared to the improvement among women. However, there is lack of studies that have considered gender difference in life satisfaction during the retirement transition.

Furthermore, previous studies have also attributed the improvement in life satisfaction during the retirement transition to the financial resources (Calasanti et al. 2021; Carr et al. 2020; Weber and Hülür 2020). A stronger improvement has been reported among the retirees who were financially stable. However, there is lack of studies considering the role of occupation, one of the important proxies for financial stability (Galobardes et al. 2006). Therefore, we want to expand the previous literature by investigating differences in life satisfaction during the retirement transition based on occupation.

Health status is likewise reported to be the significant predictor of life satisfaction among the older adults (Camacho et al. 2019; Dumitrache et al. 2017; Gana et al. 2013), and there is a two-way association between physical health and subjective well-being such as life satisfaction (Steptoe et al. 2015). Previous studies have reported small or no change in self-rated health during the transition to retirement (Mein et al. 2003; Stenholm et al. 2020), while few other studies have reported an improvement (Majer et al. 2011; Westerlund et al. 2009). However, the role of health status in life satisfaction during the retirement transition is still unclear.

Moreover, family relationships such as marital status and spousal working status may influence on life satisfaction during retirement years because the retirement transition process is crucial event in life that requires necessary adjustments in part of both spouses (Kim and Moen 2002). According to the theory of interdependence of linked lives during the life transitions, the retirement transition process has been conceptualized also as a relational transition (Kim and Moen 2001). Further, the role of employment status of partners and involvement in family and marital roles has been further emphasized as an integral aspect affecting the process of retirement transition by previous studies (Calasanti et al. 2021; Price and Joo 2005; Weber and Hülür 2020; Zang 2020). Previous studies considering the family relationship status reported that the satisfaction with life (Price and Joo 2005) and psychological well-being (Kim and Moen 2002) among married women improved more compared to single women during the retirement transition. Likewise, increased life satisfaction was reported among both retirees and their partners (Weber and Hülür 2020). What is currently lacking in the literature is how spousal working status influences on life satisfaction during the retirement transition.

To address the gaps in the literature, the aim of this longitudinal study was to examine changes in total and domain-specific life satisfaction during the retirement transition period. The domain-specific changes were examined in terms of personal feelings in relation to interestingness, happiness, easiness and togetherness. We additionally examined whether the change in total and domain-specific life satisfaction was dependent on pre-retirement characteristics that are known to associate with life satisfaction such as gender, occupations, self-rated health and spousal working status.

We build our hypothesis based on findings from previous empirical studies. We hypothesize, first, that life satisfaction (total and domain-specific) increases during the retirement transition period and the increase is higher among men than among women (Calasanti et al. 2021). Life could get easy, and retirees may feel content with their life to execute new roles (Kim and Moen 2001). Previous studies have also reported higher overall well-being among men than among women during retirement transition (Kim and Moen 2001). Second, we hypothesize that the individuals in occupation that require high skill have higher improvement in life satisfaction than among those in occupations that require low skills as previous studies have reported a stronger improvement among those with better financial status during retirement (Calasanti et al. 2021). Third, we hypothesize that the change in life satisfaction during retirement transition depends on pre-retirement health status so that those having poorer health improve more because of short-term easiness and removal of work-related stress after retirement (Gana et al. 2013). Fourth, we hypothesize that the changes of an individual’s life satisfaction during retirement transition also depend on working status of their spouse during retirement, because retirement transition period has been conceptualized as a relational transition by theory of interdependence of linked lives (Kim and Moen 2002).

## Methods

### Study population

The study population consisted of older public sector employees from the Finnish Retirement and Aging Study cohort study (FIREA), which is an ongoing longitudinal cohort study of retiring public sector workers in Finland established in 2013 (Leskinen et al. 2018; Stenholm et al. 2020). The eligible population of the FIREA study was employees whose individual retirement date was between 2014 and 2019 and who were working in the year 2012 in one of the 27 municipalities in southwest Finland or in the nine selected cities or five hospital districts across Finland. Participants were first contacted 18 months prior to their estimated individual pensionable age, obtained from the institute for public sector pensions in Finland, by sending a questionnaire, and the follow-up questionnaire was sent annually. The participants responded on average 4.3 (SD 0.8) times to the surveys. Informed consent was obtained from the participants, the ethics committee of Hospital district of Southwest Finland approved the study, and the FIREA study was conducted in line with the declaration of Helsinki.

By the end of 2019, in total 6,783 of the FIREA cohort members (64% of the eligible sample, *n* = 10,629) had responded to at least one questionnaire. This study is comprised of two possible study waves before retirement (wave − 2, wave − 1) and three possible waves after retirement (wave + 1, wave + 2, wave + 3) with each successive wave one year apart from each other. To be eligible for the analytical sample of the study cohort, the participants needed to have answered to the life satisfaction questionnaire in waves before (wave − 1) and after retirement (wave + 1) (*n* = 3543). The description of pre-retirement, retirement transition and post-retirement period with annual study waves and the study design are presented in eTable **1**. The selected participants provided information on total life satisfaction at an average of 3.8 (SD 0.6) of the possible five study waves. There were no major differences in terms of background characteristics between the final analytical sample and the eligible population (83% vs 80% of women).

### Timing of retirement for Finnish public sector employees

In Finland, the Public Sector Pensions Act regulates the retirement ages of the public sector employees. From 2005 onward, public sector employees can retire on a statutory basis after aged 63 years but at the latest before the age of 68 years. Following a pension reform in January 2017, each age group has their own retirement age, which is tied to the life expectancy, although the general rule of 63 to 68 years still applies. However, some public sector employees have chosen to keep their earlier retirement age from the previous pension act in which pension ages in some occupations were below 63 years (e.g., 60 years for primary school teachers, 58 for practical nurses). The institute for public sector pensions in Finland (Keva) has calculated an individual pensionable date for each employee accordingly. Postponing retirement from this date will accrue pension income level. (Eläketurvakeskus 2017). The average retirement age in this sample was 63.8 years (Standard deviation, 1.2).

### Assessment of life satisfaction

Life satisfaction was assessed in each study waves using a scale with four self-rated questions (Andrews and Stephen 1976), which were modified from the questionnaire that was initially developed to assess the quality of life (Allardt 1973). The scale comprises of four domains of life satisfaction, namely “interestingness,” “happiness,” “easiness” and “loneliness,” which were inquired with single questions: “Do you feel that your life at present is interesting?”, “Do you feel that your life at present is happy?”, “Do you feel that your life at present is easy?” “Do you feel that your life at present is lonely?”, respectively. There were five response alternatives, and separate ratings for each response were given, partly following procedure used in the previous study (Koivumaa-Honkanen et al. 2000). The original responses and re-ordering of responses is described in detail in eTable **2**.

The total life satisfaction score was computed by summing up the responses in four domains of life satisfaction. The sum score ranged from 4 to 20, with increasing values indicating improved life satisfaction. To harmonize the scores used in our study, the name of the domain “loneliness” has been renamed to “togetherness” henceforth. If the response was missing for three or four domains, the sum score of total life satisfaction was treated as missing. In addition, we used domain-specific life satisfaction information and the domain-specific scores ranged from 1 to 5 with increasing values indicating improved domain-specific life satisfaction. The calculation of life satisfaction based on these four domains was validated and used in a previous study (Koivumaa-Honkanen et al. 2000).

### Assessment of pre-retirement characteristics

Information on participant’s sex, date of birth and occupational titles was obtained from the institute for public sector pensions in Finland. Before the retirement transition, the mean age of the study population was 63.4 years (standard deviation 1.4). The occupational titles of the last occupation preceding retirement were coded according to the International Standard Classification of Occupations (ISCO-08). We classified occupations according to the broad skill levels in three categories: skill levels 3 and 4 (ISCO-08 classes 1–3: e.g., managers, professionals), skill level 2 (ISCO-08 classes 4–8: e.g., clerical support workers, service sale workers) and skill level 1 (ISCO-08 class 9: elementary occupations) (International Labour Organization, 2008). As there were very few people in skill level 1, we merged skill level 1 and skill level 2 that makes it two occupational categories (*high*: skill level 3 and 4 and *low*: skill level 1 and 2) for the analytical purpose. The other pre-retirement characteristics were obtained from the survey preceding retirement (wave − 1). *Marital status* was originally collected in five categories (never married, cohabitation, married, divorced or separated and widowed), and in order to use in this study, it was dichotomized into currently married/cohabiting (yes) and non-married/non-cohabiting (no). *Spousal working status* was created based on the marital status of respondents (wave − 1), working status of their spouses one year before retirement (wave − 1) and working status of their spouses one year after retirement (wave + 1). We created three categories of spousal working status namely “*No spouse*: never married, divorced or separated and widowed,” “*Working *(*-1,* + *1*): spouse working full time at both time points (wave − 1) and (wave + 1)” and “*Retired (*− *1,* + *1)*: spouse retired at both time points (wave − 1) and (wave + 1).”

*Self-rated health* was assessed by asking participant to rate their overall health status on a 5-point scale (1—“good,” 2—“rather good,” 3—“average,” 4—“rather poor” and 5—“poor”). The responses were dichotomized into good (“good” and “rather good”) and suboptimal (“average,” “rather poor” and “poor”). We used dichotomized self-rated health as it is commonly used in previous studies (Stenholm et al. 2020).

### Statistical analysis

We used frequencies and percentage for categorical and means and standard deviation (SD) for continuous variables to present the pre-retirement characteristics (at wave − 1) of the study population. To illustrate the level of total and domain-specific life satisfaction across retirement transition, we first calculated the mean estimates and their 95% confidence intervals (CI) in each of the study waves (wave − 2 to wave + 3) by using linear regression models with generalized estimating equations (GEEs). The advantages of using the GEEs over other contemporary models is that GEEs are not sensitive to measurements missing completely at random and control for the intra-individual correlation between repeated measurements using an exchangeable correlation structure (Diggle et al. 1994; Zeger et al. 1988).

To study changes in total and domain-specific life satisfaction during retirement transition, we created retirement transition period from wave − 1 to wave + 1 corresponding approximately 0.5 years before and 0.5 years after retirement. The change in total and domain-specific life satisfaction during retirement transition period (wave − 1 to wave + 1) is presented as mean change and their 95% CI. The mean estimates and mean change during the retirement transition period (wave − 1 to wave + 1) are presented in two different models: first adjusted for age, gender, and occupational categories (model I), and second, additionally adjusted for self-rated health and marital status (model II), except for estimates for spousal working status (model II: additionally adjusted for self-rated health).

Finally, we examined whether the changes in total and domain-specific life satisfaction differ by pre-retirement characteristics such as gender, occupational categories, self-rated health and spousal working status. For these analyses, the interaction term pre-retirement characteristic*time was added to the GEEs and are presented in two different models: first adjusted for age, gender and occupational categories (model I) and second, additionally adjusted for self-rated health and marital status (model II), except for estimates for spousal working status (model II: additionally adjusted for self-rated health). The effect size is presented as standardized mean difference (SMD) which is the ratio of mean change and standard deviation (SD) (SMD = mean change/SD) (Andrade 2020). The SAS V.9.4 Statistical Package was used for the analyses (SAS Institute).

## Results

The pre-retirement (at wave − 1) characteristics of the study population and the level of total and domain-specific life satisfaction (mean, SD) at wave − 1 according to these characteristics are shown in Table 1. More than 80% of the study population were women. Majority of our study population belonged to high-skill level occupations (58%). One fourth of the study population (24%) had suboptimal self-rated health before retirement. A little more than one fourth (27%) had no spouse and of the married participants, 14% had working spouse, while spouses of 59% were already retired during both time points (wave − 1 and wave + 1). There were no differences in level of total and domain-specific life satisfaction based on gender and occupational categories at wave − 1. The level of total life satisfaction was higher among those with good (mean 16.9, SD 2.3) than those with suboptimal (mean 15.2, SD 3.1) self-rated health, and the level of each of the domain-specific life satisfaction at wave − 1 was similarly higher among those with good self-rated health. Those who had either working (mean 16.9, SD 2.4) or retired (mean 16.8, SD 2.3) spouse had higher level of total life satisfaction than those who had no spouse (mean 15. 7, SD 3.1), and these distributions were similar for level of domain-specific life satisfaction.

**Table 1:** Mean (standard deviation) of total and domain-specific life satisfaction scores before retirement (wave − 1) by pre-retirement characteristics of the study population

Pre-retirement characteristics	*n*	%	Life satisfaction score before retirement (w − 1), Mean (SD)				
			Total	Domains			
				Interesting	Happiness	Easiness	Togetherness
Total	3543	100	16.5 (2.6)	4.2 (0.8)	4.1 (0.7)	3.8 (0.9)	4.5 (1.1)
*Gender*							
Women	2947	83	16.5 (2.7)	4.2 (0.8)	4.1 (0.7)	3.8 (0.9)	4.5 (1.1)
Men	596	17	16.6 (2.5)	4.1 (0.7)	4.1 (0.6)	3.9 (0.8)	4.5 (1.1)
*Occupational category*							
High skill	2042	58	16.5 (2.6)	4.2 (0.8)	4.1 (0.7)	3.8 (0.9)	4.5 (1.1)
Low skill	1501	42	16.5 (2.7)	4.1 (0.8)	4.1 (0.7)	3.9 (0.8)	4.5 (1.1)
*Self-rated health*							
Good	2693	76	16.9 (2.3)	4.3 (0.7)	4.2 (0.6)	3.9 (0.8)	4.6 (1.0)
Suboptimal	843	24	15.2 (3.1)	3.8 (0.8)	3.8 (0.8)	3.5 (1.0)	4.2 (1.3)
*Spousal working status*							
No spouse	916	27	15.7 (3.1)	4.0 (0.9)	4.0 (0.7)	3.7 (0.9)	4.0 (1.4)
Working (− 1, + 1)	458	14	16.9 (2.4)	4.2 (0.8)	4.2 (0.7)	3.9 (0.9)	4.6 (1.0)
Retired (− 1, + 1)	1970	59	16.8 (2.3)	4.2 (0.7)	4.2 (0.6)	3.9 (0.8)	4.7 (0.9)

SD, Standard Deviation; w − 1, year before retirement; *Spousal working status*: “*No spouse*: never married, divorced or separated and widowed,” “*Working (*− *1,* + *1)*: spouse working full time at both time points wave − 1 and wave + 1” and “*Retired (*− *1,* + *1)*: spouse retired at both time points wave − 1 and wave + 1”

**Fig. 1:**
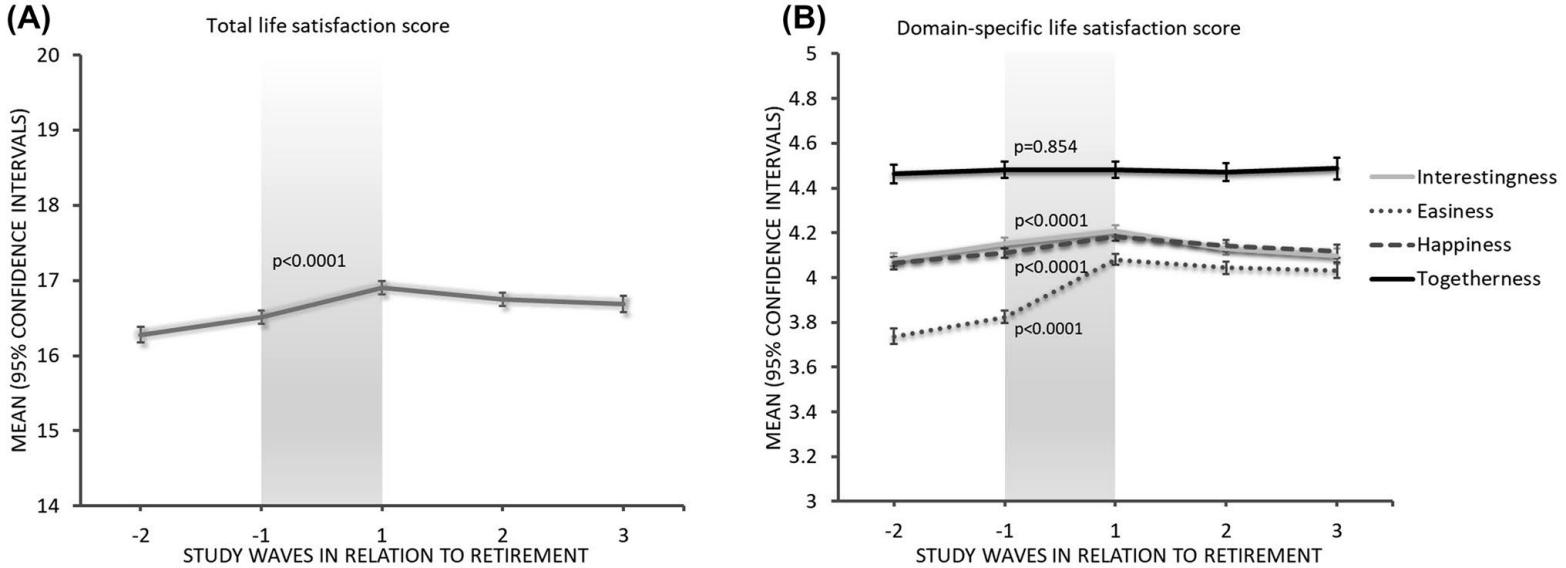
Mean (95% CI) total life satisfaction scores (**A**) and domain-specific life satisfaction scores (**B**) in overall population before, during and after retirement; waves − 1 to + 1 indicate retirement transition period (study waves are one year apart from each other); *p* value in panel A and panel B is for change during retirement transition

**Fig. 2:**
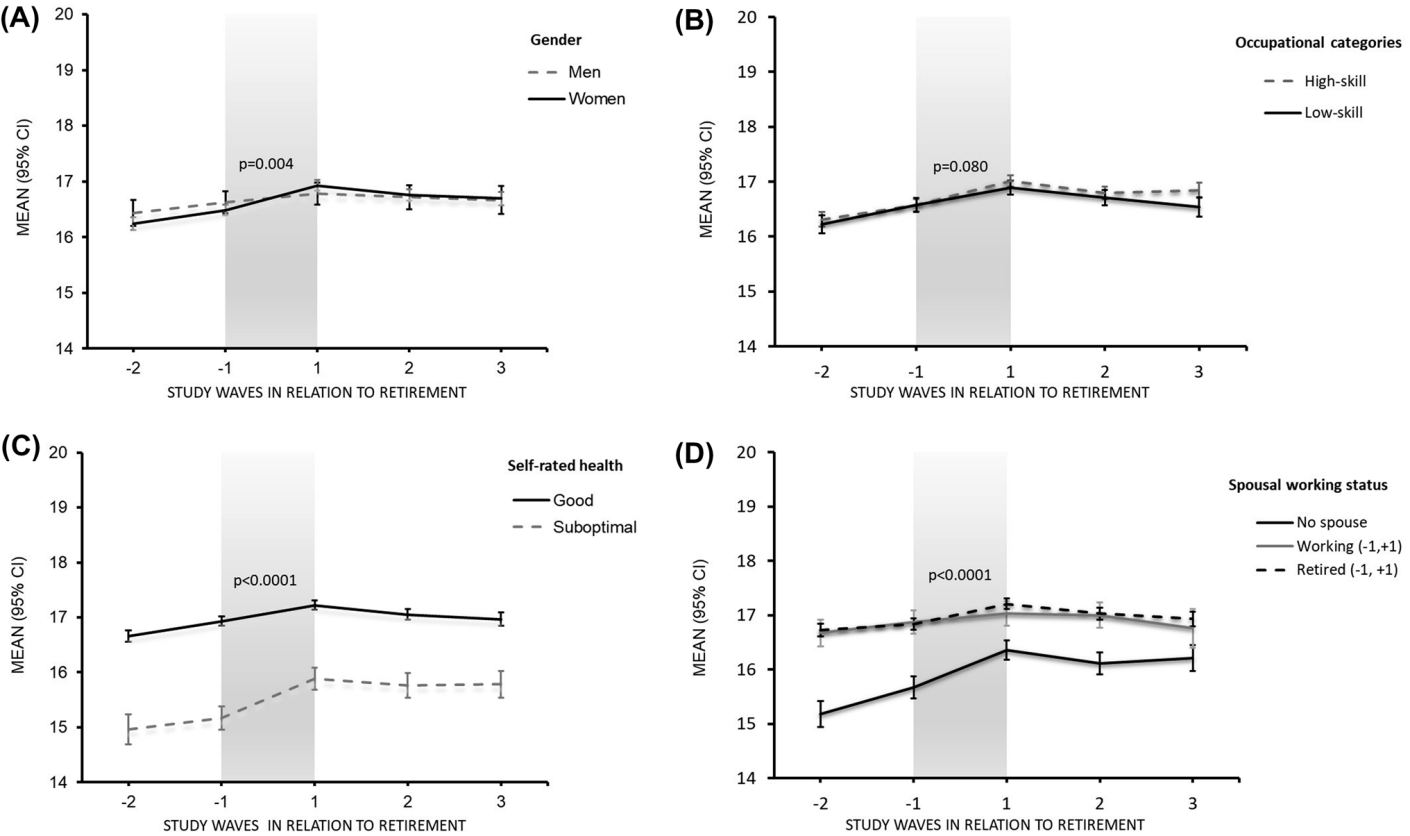
Mean (95% CI) total life satisfaction scores before, during and after retirement based on gender (**A**), occupation (**B**), self-rated health (**C**) and spousal working status (**D**); Waves − 1 to + 1 indicate retirement transition period (study waves are one year apart from each other); *p *values are for interaction of pre-retirement characteristics with time; *spousal working status*: “*No spouse*: never married, divorced or separated and widowed,” “*Working (*− *1,* + *1)*: spouse working full time at both time points wave − 1 and wave + 1” and “*Retired (*− *1,* + *1)*: spouse retired at both wave − 1 and wave + 1”

Figure 1 illustrates the mean and 95% CI of total (A) and domain-specific (B) life satisfaction in each of the study waves (wave − 2 to wave + 3) in the total study population. There was a significant increase in total and domain-specific life satisfaction scores across the retirement transition (wave − 1 to + 1) period (*p* < 0.0001), except for the togetherness domain (*p* = 0.99). The total life satisfaction scores in each of the study waves (wave − 2 to wave + 3) based on pre-retirement characteristics of the study population are presented in Fig. 2 in four different panels (A) Gender, (B) Occupational categories, (C) Self-rated health and (D) Spousal working status. There was a significant difference in increase in total life satisfaction based on gender (p = 0.004, gender*time), self-rated health (*p* < 0.0001, self-rated health*time) and spousal working status (*p* < 0.0001, spousal working status*time) except for occupational categories (p = 0.0804, occupation*time) during the retirement transition period. The Online supplementary figures: eFigure **1** (gender), eFigure **2** (occupation), eFigure **3** (self-rated health) and eFigure **4** (spousal working status) present the mean and 95% CI of domain-specific life satisfaction in each of the study waves (wave − 2 to wave + 3). There was a significant difference in increase in interestingness and easiness domains based on gender and self-rated health, and the increase in happiness was significant for gender, self-rated health, and spousal working status during the retirement transition. However, the increase in togetherness domain was significant for spousal working status only.

The total life satisfaction scores (mean estimate and 95% CI) before retirement and mean change during the retirement transition (wave − 1 to wave + 1) and standardized mean difference (SMD) for change is presented in Table 2. Before retirement (at wave − 1), the mean total life satisfaction score adjusted for age, gender, occupation, self-rated health and marital status was 15.8 (95% CI 15.7–16.0), which was increased during retirement transition with a corresponding mean change of 0.5 (95% CI 0.4–0.6). The effect size for change in total life satisfaction during retirement transition was small (SMD = 0.19). In case of domains (Table 3), the highest change in adjusted mean scores during retirement transition was observed in easiness (mean change 0.27; 95% CI 0.23–0.30) (SMD = 0.3) followed by happiness (0.09; 0.06–0.11) (SMD = 0.13) and interestingness (0.05; 0.02–0.08) (SMD = 0.06), and the least change was observed in togetherness domain (0.04; 0.003–0.09) (SMD = 0.04).

**Table 2:** Total life satisfaction scores (mean estimate and 95% CI) before retirement and changes (mean change and 95% CI) during the retirement transition period (wave − 1 to wave + 1) by pre-retirement characteristics of the study population

Pre-retirement characteristics	Mean (95% CI)				Mean change (95% CI)				
	Before retirement (wave − 1)				Retirement transition (wave − 1 to wave + 1)				
	Model I^a^	*P* value^ǂ^	Model II^b^	*P* value^ǂ^	Model I^a^	*P* value^*^	Model II^b^	*P* value^*^	SMD
Total	16.5 (16.4, 16.7)		15.9 (15.7, 16.0)		0.4 (0.3, 0.5)		0.5 (0.4, 0.6)		0.19
*Gender*		0.223		0.709		0.004		0.004	
Women	16.5 (16.4, 16.6)		15.8 (15.7, 15.9)		0.5 (0.4, 0.6)		0.5 (0.4, 0.6)		0.19
Men	16.6 (16.4, 16.9)		15.9 (15.7, 16.1)		0.2 (−0.0, 0.4)		0.2 (0.0, 0.4)		0.08
*Occupational category*		0.974		0.023		0.160		0.161	
High skill	16.6 (16.4, 16.7)		15.8 (15.6, 15.9)		0.5 (0.4, 0.6)		0.5 (0.4, 0.6)		0.19
Low skill	16.6 (16.4, 16.7)		16.0 (15.8, 16.1)		0.4 (0.2, 0.5)		0.4 (0.3, 0.5)		0.15
*Self-rated health*		< 0.0001		< 0.0001		< 0.0001		< 0.0001	
Good	17.0 (16.9, 17.2)		16.7 (16.6, 16.8)		0.4 (0.3, 0.5)		0.4 (0.3, 0.4)		0.17
Suboptimal	15.2 (15.0, 15.4)		14.9 (14.7, 15.2)		0.9 (0.7, 1.0)		0.8 (0.6, 1.0)		0.26
*Spousal working status*		< 0.0001		< 0.0001		< 0.0001		< 0.0001	
No spouse	15.6 (15.5, 15.8)^c^		15.3 (15.1, 15.5)^c^		0.7 (0.5, 0.8)		0.7 (0.6, 0.9)^c^		0.23
Working (− 1, + 1)	16.9 (16.6, 17.1)^c^		16.4 (16.1, 16.6)^c^		0.1 (−0.1, 0.4)		0.2 (−0.0, 0.4)^c^		0.08
Retired(− 1, + 1)	16.8 (16.7, 17.0)^c^		16.4 (16.3, 16.6)^c^		0.3 (0.2, 0.4)		0.4 (0.3, 0.5)^c^		0.17

CI Confidence Interval, SMD Standardized mean difference; − 1, year before retirement and + 1, year after retirement

^a^ models adjusted for age, gender and occupation

^b^models adjusted for age, gender, occupation, self-rated health and marital status

^c^models adjusted for age, gender, occupation and self-rated health

^ǂ^p values for group difference, *p values for interaction of pre-retirement characteristics with time; *spousal working status*: “*No spouse*: never married, divorced or separated and widowed,” “*Working (*− *1,* + *1)*: spouse working full time at both time points wave − 1 and wave + 1” and “*Retired (*− *1,* + *1)*: spouse retired at both time points wave − 1 and wave + 1”

**Table 3:** Changes (mean change and 95% CI) in domain-specific life satisfaction score during the retirement transition period (wave − 1 to wave + 1) by pre-retirement characteristics of the study population

Pre-retirement	Mean change (95% CI) during retirement transition											
Characteristics	Interestingness^a^	*P* value^*^	SMD	Happiness^a^	*P* value^*^	SMD	Easiness^a^	*P* value^*^	SMD	Togetherness^a^	*P* value^*^	SMD
Total	0.05 (0.02, 0.08)		0.06	0.09 (0.06, 0.11)		0.13	0.27 (0.23, 0.30)		0.3	0.04 (0.003, 0.09)		0.04
*Gender*		0.022			0.004			0.012			0.457	
Women	0.07 (0.03, 0.10)		0.09	0.10 (0.07, 0.13)		0.14	0.28 (0.25, 0.32)		0.3	0.05 (0.007, 0.09)		0.05
Men	−0.02 (−0.08, 0.05)		−0.03	0.02 (−0.03, 0.07)		0.03	0.19 (0.13, 0.26)		0.24	0.02 (−0.07, 0.109)		0.02
*Occupational category*		0.918			0.175			0.978			0.059	
High skill	0.05 (0.02, 0.09)		0.06	0.10 (0.07, 0.13)		0.14	0.27 (0.23, 0.31)		0.3	0.07 (0.02, 0.12)		0.06
Low skill	0.05 (0.01, 0.09)		0.06	0.07 (0.03, 0.11)		0.1	0.27 (0.22, 0.31)		0.34	0.005 (−0.05, 0.06)		0.01
*Self-rated health*		0.003			0.006			< 0.0001			0.668	
Good	0.03 (−0.004, 0.06)		0.04	0.07 (0.04, 0.09)		0.12	0.22 (0.19, 0.25)		0.28	0.04 (−0.004, 0.08)		0.04
Suboptimal	0.13 (0.07, 0.20)		0.16	0.15 (0.10, 0.20)		0.19	0.42 (0.35, 0.49)		0.4	0.06 (−0.02, 0.14)		0.05
*Spousal working status*		0.454			0.013			0.609			< 0.0001	
No spouse	0.07 (0.02, 0.13)^b^		0.08	0.14 (0.09, 0.18)^b^		0.2	0.29 (0.23, 0.35)^b^		0.32	0.19 (0.11, 0.27)^b^		0.14
Working (− 1, + 1)	0.02 (−0.05, 0.10)^b^		0.03	0.04 (−0.02, 0.10)^b^		0.06	0.24 (0.16, 0.32)^b^		0.27	−0.07 (−0.16, 0.03)^b^		−0.07
Retired(− 1, + 1)	0.05 (0.01, 0.08)^b^		0.07	0.07 (0.05, 0.10)^b^		0.12	0.26 (0.23, 0.30)^b^		0.32	−0.002 (−0.05, 0.04)^b^		0

CI Confidence Interval; SMD Standardized mean difference; − 1, year before retirement and + 1, year after retirement

^a^ models adjusted for age, gender, occupation, self-rated health and marital status

^b^models adjusted for age, gender, occupation and self-rated health

*p values for interaction of pre-retirement characteristics with time; *spousal working status*: “*No spouse*: never married, divorced or separated and widowed),” “*Working (*− *1,* + *1)*: spouse working full time at both time points wave − 1 and wave + 1” and “*Retired (*− *1,* + *1)*: spouse retired at both time points wave − 1 and wave + 1”

### Change in life satisfaction by gender

There was no gender difference in the total life satisfaction score before retirement. However, life satisfaction increased more among women than among men during the retirement transition when fully adjusted (mean change 0.5, 95% CI 0.4–0.6 vs. 0.2, 95% CI 0.0–0.4, gender*time interaction p = 0.004, SMD = 0.19) for age, occupation, self-rated health and marital status (Table 2). Before retirement, the significant difference was observed only in easiness domain based on gender (*p* = 0.0006), with satisfaction being higher among men than among women (eTable**3**). The gender difference was observed in change in interestingness, happiness and easiness domains (gender*time interaction *p* < 0.05) across the retirement transition but not in the change in togetherness domain. The change in all three domains during the retirement transition was greater among women compared to men, and marked difference was observed in easiness domain (mean change 0.28, 95% CI 0.25–0.32 vs. 0.19, 95% CI 0.13–0.26, gender*time interaction *p* = 0.004, SMD = 0.3) (Table 3).

### Change in life satisfaction by occupation

The total life satisfaction before retirement differed by occupational categories (*p* = 0.023), with higher satisfaction among those in high-skill level than among those in low-skill level occupations. However, there was no difference in change in total life satisfaction during the retirement transition based on occupational categories (mean change 0.5, 95% CI 0.4–0.6 vs. 0.4, 95% CI 0.3–0.5, occupation*time interaction *p* = 0.1610) in fully adjusted model (Table 2). The significant difference based on occupational categories (*p* < 0.05) was observed in easiness and togetherness domains before retirement with higher satisfaction among those in low-skill level than among those in high-skill level occupation (eTable**3**). There was no difference in change in all four domains of life satisfaction during the retirement transition period based on occupational categories (Table 3).

### Change in life satisfaction by self-rated health

The total life satisfaction score adjusted for age, gender, occupation, self-rated health, and marital status before retirement (wave − 1) differed by self-rated health and was higher for those who reported good self-rated health (mean 16.7, 95% CI 16.6–16.8) compared to those with suboptimal self-rated health (mean 14.9, 95% CI 14.7–15.2). However, the total life satisfaction among those with suboptimal self-rated health before retirement was markedly increased during the retirement transition period compared to those who had good self-rated health before retirement (mean change 0.8, 95% CI 0.6–1.0, SMD = 0.26 vs. 0.4, 0.3–0.4, SMD = 0.17, self-rated health*time interaction *p* < 0.0001) when fully adjusted (Table 2). Before retirement, the significant difference was observed in every domain-specific life satisfaction based on self-rated health (*p* < 0.0001), with satisfaction in every domain being higher for those with good self-rated health (eTable**3**). Further, the self-rated health difference in change across the retirement transition was significant for interestingness, happiness and easiness domains (self-rated health*time interaction *p* < 0.05) with marked changes in easiness domain that was greater among those with suboptimal (0.42, 0.35–0.49, SMD = 0.4) than among those with good (0.22, 0.19–0.25, SMD = 0.28) self-rated health (Table 3).

### Change in life satisfaction by spousal working status

The total life satisfaction before retirement (wave − 1) was higher for those with a spouse irrespective of their spouse’s working status (mean 16.4, 95% CI 16.3–16.6) compared to those who had no spouse (*p* < 0.0001). The change in life satisfaction during retirement transition based on spousal work status was significant with higher increase among those who had no spouse (mean change 0.7, 95% CI 0.6–0.9, SMD = 0.233) than among the married/co-habited respondents irrespective of their spouse’s working status (spousal working status*time interaction *p* < 0.0001) when fully adjusted (Table 2). The significant difference based on spousal working status (*p* < 0.0001) was observed in all four domain-specific life satisfaction before retirement with higher satisfaction level in every domain for those whose spouse was retired (eTable**3**). However, the change across the retirement transition was significant for togetherness domain only (spousal working status*time interaction *p* < 0.0001) with a greater improvement for those who had no spouse (0.19, 0.11–0.27, SMD = 0.17) than among those who were married/co-habited irrespective of their spouse’s working status (Table 3).

## Discussion

To our knowledge, this is one of the first prospective studies examining changes in both total and domain-specific life satisfaction across the retirement transition. Life satisfaction in general improved during the retirement transition and stabilized thereafter. Likewise, there was an improvement in interestingness, easiness and happiness domains of life satisfaction during the retirement transition, whereas the togetherness domain remained constant. During the transition to retirement, life satisfaction improved more among women, among those with suboptimal self-rated health and among those who had no spouse with a small effect size. The improvement was greater in the easiness domain of life satisfaction than other domains, and it was greater for those with suboptimal health than for those with good health and greater for those having no spouse than for those having spouse irrespective of their spouse’s working status.

Our findings are in agreement with the previous longitudinal studies suggesting that life satisfaction improves during the retirement transition (Calasanti et al. 2021; Carr et al. 2020; Gorry et al. 2018; Hershey and Henkens 2014; Weber and Hülür 2020). An advantage of our study over the previous studies is the investigation of annual change in life satisfaction using the measures of both total and domain-specific life satisfaction scores. The domain-specific changes were examined in terms of personal feelings in relation to interestingness, happiness, easiness and togetherness, whereas previous study using the HRS cohort from the US examined life satisfaction in terms of leisure, family and finance (Calasanti et al. 2021). In addition, the study population in our study was relatively homogenous compared to heterogeneous study populations in most of the previous studies. Homogeneity in the study population minimizes and controls for selection bias. Further, we studied the changes based on several individual and circumstantial factors such as gender, occupational status, self-rated health and spousal working status. Most of the observed changes in life satisfaction during retirement transition were marginal, and the effect size for change was small because the life satisfaction was already at quite high level among our study participants before retirement. However, a small difference could be considered better and meaningful than no difference in terms of effect size if it is presented as standardized mean difference (Andrade 2020).

The consideration of individual factor such as gender is important to understand the effects of retirement on life satisfaction (Kim and Moen 2002; Price and Joo 2005). We found firstly, that although both men and women had similar levels of life satisfaction before retirement and both had an improvement in their life satisfaction scores during the retirement transition, life satisfaction among women increased more than among men. However, Calasanti et al. (2021) reported in their study a greater improvement in life satisfaction among men than among women during the retirement transition. Although the retirement ages were rather similar between our and their study (Calasanti et al. 2021), our study was larger and female-dominated compared to their study, which hinders comparability of these two studies.

Previous studies have attributed the improvement in life satisfaction during the retirement transition to financial resources (Calasanti et al. 2021; Carr et al. 2020; Weber and Hülür 2020). Kim & Moen (2002) used the cohort from US in their study and reported the important role of financial situation for the changes in well-being during the retirement transition (Kim and Moen 2002). Similarly, the improved life satisfaction among men compared to women during the transition period was associated with lower financial satisfaction among women in the HRS cohort (Calasanti et al. 2021). In addition, retirees with higher financial resources reported improved life satisfaction in a study of nationally representative sample of German households (Weber and Hülür 2020). Likewise, another study using the HRS cohort reported that financially vulnerable retirees were more likely to report decreased life satisfaction during the retirement transition compared to their financially rich and stable counterparts (Carr et al. 2020). We considered occupation as a proxy for financial status and examined differences in life satisfaction between occupations categorized according to broad skill level. We found that those in high-skill level were more satisfied before retirement; however, the improvement in life satisfaction during the retirement transition was similar in both high- and low-skill level occupations. These results are in contrast with the previous findings where retirees with higher financial resources reported improved life satisfaction during retirement transition (Hershey and Henkens 2014). These differences may be due to the lack of direct measurement of financial resources in our study. However, a decline in financial status could come as a contextual change during the retirement transition (Hershey and Henkens 2014), resource-rich individuals are less likely to experience retirement-related changes (Pinquart and Schindler 2007), and the level of satisfaction could differ based on the type of retirement.

Since health status may influence life satisfaction (Camacho et al. 2019; Dumitrache et al. 2017; Gana et al. 2013; Steptoe et al. 2015), we were interested in investigating the change in life satisfaction during the transition period based on self-rated health before retirement, which was found to be an important explanatory factor of life satisfaction. In line with the previous study (Gana et al. 2013), we found life satisfaction was higher among those with good self-rated health than among those with suboptimal self-rated health throughout the follow-up period. However, the improvement in life satisfaction during the retirement transition period was greater among those with suboptimal self-rated health than among those with good self-rated health before retirement. Since retirement comes as a relief from work-related stress and more easiness (Atchley RC 1976), the people with poor health feel more satisfied with their life and tend to improve more in their life satisfaction levels compared to their healthier counterparts who already have a quite higher level of life satisfaction before retirement.

Further, the importance of spousal working status during retirement transition has been highlighted by previous studies (Calasanti et al. 2021; Kim and Moen 2002), which motivated us to study the change in life satisfaction during retirement transition by spousal working status. A previous study focused on spousal working status as a predictor of change in well-being during retirement transition and found that there was no difference in well-being depending on spousal working status (Kim and Moen 2002). Similar to the findings from the previous study (Kim and Moen 2002), we did not find any difference in change in life satisfaction based on spousal working status. However, life satisfaction among those who had no spouse remained lower than among those who had spouse throughout the follow-up period, which is consistent with the previous finding (Price and Joo 2005). Moreover, the improvement in life satisfaction during the retirement transition period was greater among those who had no spouse than among married/ cohabiting respondents with different spousal working status. The improvement in life satisfaction was discontinued thereafter, and it was constant during the post-retirement period. This could be explained by the fact that retirement comes with short-term easiness as life satisfaction improves during the retirement transition process, and it remains constant during post-retirement phase as a person adapts the new situation.

Regarding the domain-specific findings of our study, more marked improvement was observed in the easiness domain than among other domains, partly explained by the feeling of easiness as a person nears the retirement period (Atchley RC 1976; Kim and Moen 2001). Most of the observed changes in our study were stable, as the improved life satisfaction during the transition phase remained during the post-retirement phase. During the retirement transition period, the retirees may feel content with their life with an increased level of energy to execute the new roles in society and to plan desired activities in post-retirement life, which could be attributed to the concept of “honeymoon phase” (Atchley RC 1976). Moreover, the retirees may be relieved from the pressure of the jobs, and they could enjoy the short-term well-being as a part of retirement relief that could be partly related to the role strain hypothesis (Kim and Moen 2001, 2002). The removal of work-related schedules as well as physical and psychosocial work stressors (e.g., strenuous working conditions, high demands, low appreciation) and availability of more sleeping time (Myllyntausta et al. 2017) come with retirement. In addition, there are more opportunities and time to be involved in leisure activities and other social activities after retirement (Heaney et al. 2014; Henning et al. 2021; Stenholm et al. 2016; Vigezzi et al. 2021). Thus, retirement may be experienced as a gain and relief, and this may be reflected as an increased feelings of easiness during the transition to retirement. Moreover, we and others have shown earlier that psychological distress decreases (Lahdenperä et al. 2022) and self-rated health improves (Stenholm et al. 2021; Westerlund et al. 2009) after retirement transition, which could partly explain increased easiness among those who had suboptimal self-rated health before retirement in our study. Further, part of our findings could be explained by the financial aspects as the earned accrued pension rights in Finland offers relatively rewarding pension schemes for Finnish old-age pensioners (Eläketurvakeskus 2017; Palomäki et al. 2022). However, this question was not examined in the current study.

The major strength of this study is the use of repeated yearly measurements for a comprehensive period among a relatively homogenous working population over the transition to statutory retirement. The availability of information on actual retirement age is another strength of this study. When first contacted, all the respondents were still in employment, which partly controlled for health-related selection bias.

The dependence on self-rated life satisfaction scores could be a subject to bias and estimation error in ratings; however, life satisfaction is an indicator of a general well-being assessed through anticipated subjective feeling of an individual (Moen 1996), and self-rated items are the only means to measure subjective feelings such as well-being and life satisfaction. Moreover, as we used repeated measurements, the participant served as their own controls, effectively controlling for individual differences in response style. The other possible limitation of this study is that we calculated the total life satisfaction score based on domain-specific scores, and we were not able to control the simultaneity of domain-specific life satisfaction, which could have resulted in slight overestimation of total life satisfaction score. However, this way of calculation of total life satisfaction has been previously validated and used (Koivumaa-Honkanen et al. 2000; Korpimäki et al. 2012). Likewise, the information on domain-specific life satisfaction in each of the five study waves were gathered using the same questionnaire.

Furthermore, the study population of this study is a representative sample of public sector employees in Finland; therefore, the findings may not necessarily be generalizable to other sectors. In addition, the results should be cautiously generalized to male workers, since majority of our participants were women. However, the gender distribution in our study (83% women) is typical in Finnish public sector occupations in recent decades as 78% of people working in public sectors are women (Statistics Finland 2016).

## Conclusions

Life satisfaction improves during the retirement transition period as life gets easier after retirement with no work-related stress. The improvement was considerably greater among women, those with suboptimal self-rated health and those without a spouse before retirement. In case of domain-specific life satisfaction, more marked improvement was observed in the easiness domain than in other domains. The improvement remained stable during the post-retirement period suggesting that the retirees may feel content with their life with an increased level of energy to execute the new roles.

## Supplementary Information

Below is the link to the electronic supplementary material.

Supplementary file1 (PDF 555 kb)
