# Supplementary material for: Changes in life satisfaction during the transition to retirement: findings from the FIREA cohort study
Source: Eur J Ageing. 2022 Nov 23;19(4):1587–99. doi: 10.1007/s10433-022-00745-8 (PMC9729489; doi:10.1007/s10433-022-00745-8)
Supplement: Supplementary file 1 — Supplementary file1 (PDF 555 kb) [file 10433_2022_745_MOESM1_ESM.pdf]

Supplement files

Changes in life satisfaction during the transition to retirement: Findings from the FIREA cohort study

Prakash KC, PhD[1,2,3,4*], Marianna Virtanen, PhD[5,6], Soili Törmälehto, PhD[5], Saana Myllyntausta, PhD[7], Jaana Pentti, BSc[3,8,9], Jussi Vahtera, MD, PhD[3,8], Sari Stenholm, PhD[3,8]

[1]Unit of health sciences, faculty of social sciences, Tampere University, Finland

[2]Gerontology Research Center, Tampere University, Finland

[3]Department of Public Health, University of Turku and Turku University Hospital, Turku, Finland

[4]Stress Research Institute, Department of Psychology, Stockholm University, Sweden

[5]School of Educational Sciences and Psychology, University of Eastern Finland, Joensuu, Finland

[6]Division of Insurance Medicine, Karolinska Institutet, Stockholm, Sweden

[7]Department of Psychology and Speech-Language Pathology, University of Turku, Turku, Finland

[8]Centre for Population Health Research, University of Turku and Turku University Hospital; Turku, Finland

[9]Clinicum, Faculty of Medicine, University of Helsinki, Finland

[*]Corresponding author
Prakash K.C., Ph.D.,prakashkc10@gmail.com ; Prakash.kc@tuni.fi

eTable 1: Annual study waves and the study design

**Pre-retirement period**	**Retirement transition period**			**Post-retirement period**	
n=2,160<br>wave −2	n=3,543<br>wave −1	Retirement	n=3,543<br>wave +1	n=2,838<br>wave +2	n=1,433<br>wave +3

Waves around retirement period and the classification of pre-retirement period, retirement transition period and post-retirement period of the study

eTable 2: Assessment of domains of life satisfaction

Questions (domains)		Original responses	Re-ordered responses
1.	Do you feel that your life at present is interesting? (*interestingness)*	(1)-Very interesting<br>(2)-Pretty interesting<br>(3)-Pretty boring<br>(4)-Very boring<br>(5)-I do not know	(5)-Very interesting<br>(4)-Pretty interesting<br>(3)-I do not know<br>(2)-Pretty boring<br>(1)-Very boring
2.	Do you feel that your life at present is happy? (*happiness*)	(1)-Very happy<br>(2)-Pretty happy<br>(3)-Pretty sad<br>(4)-Very sad<br>(5)-I do not know	(5)-Very happy<br>(4)-Pretty happy<br>(3)-I do not know<br>(2)-Pretty sad<br>(1)-Very sad
3.	Do you feel that your life at present is easy? (*easiness*)	(1)-Very easy<br>(2)-Pretty easy<br>(3)-Pretty hard<br>(4)-Very hard<br>(5)-I do not know	(5)-Very easy<br>(4)-Pretty easy<br>(3)-I do not know<br>(2)-Pretty hard<br>(1)-Very hard
4.	Do you feel that your life at present is lonely? (*loneliness* renamed as *togetherness*)	(1)-Very lonely<br>(2)-Pretty lonely<br>(3)-I am not lonely<br>(4)-I do not know	(5)-I am not lonely<br>(3)-I do not know<br>(2)-Pretty lonely<br>(1)-Very lonely

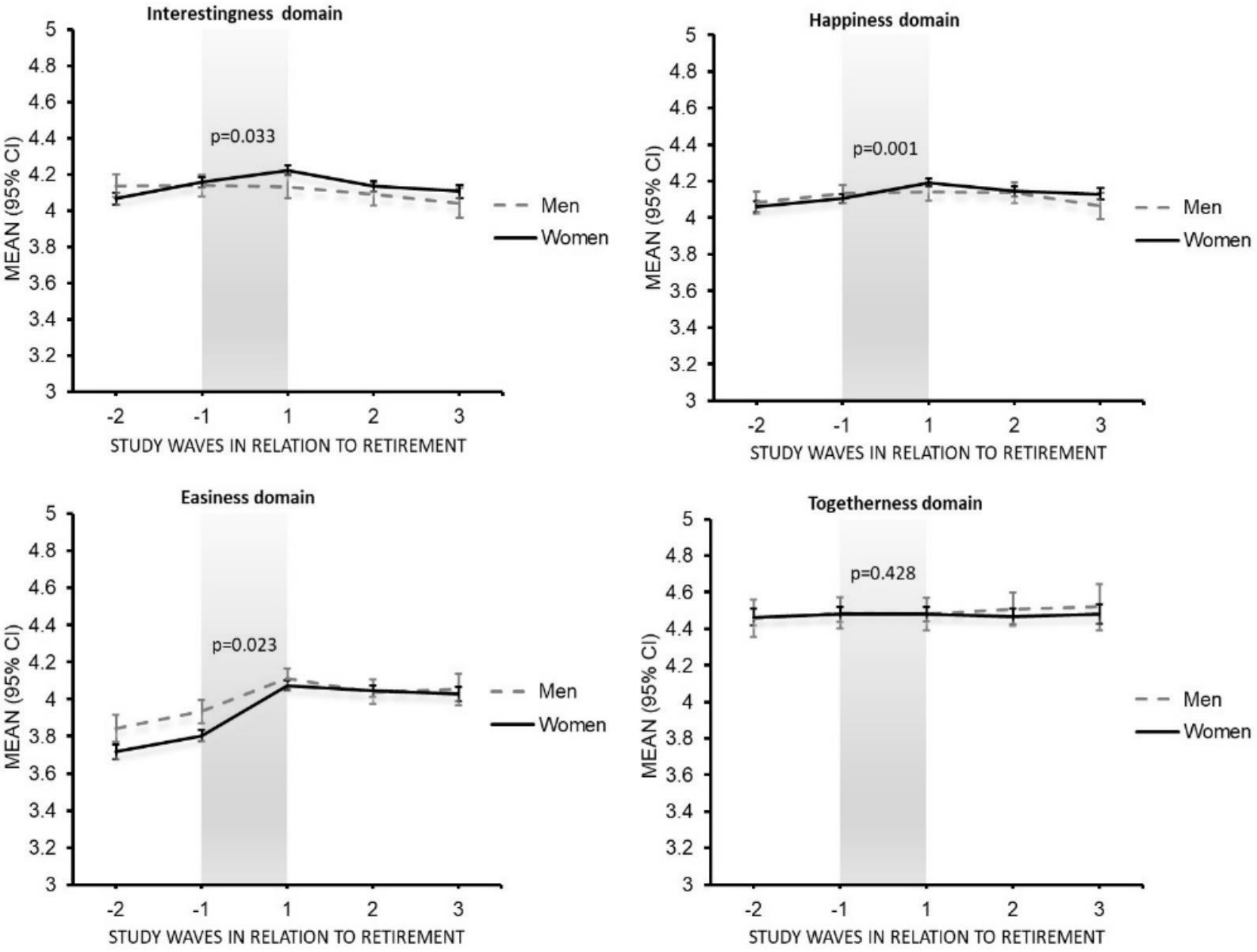

eFigure 1: Mean (95% CI) domain specific life satisfaction scores (score: 1-5) before retirement, during retirement transition and after retirement **based on gender**; p-values for interaction of gender with time; waves −1 to +1 indicate retirement transition period (study waves are one year apart from each other)

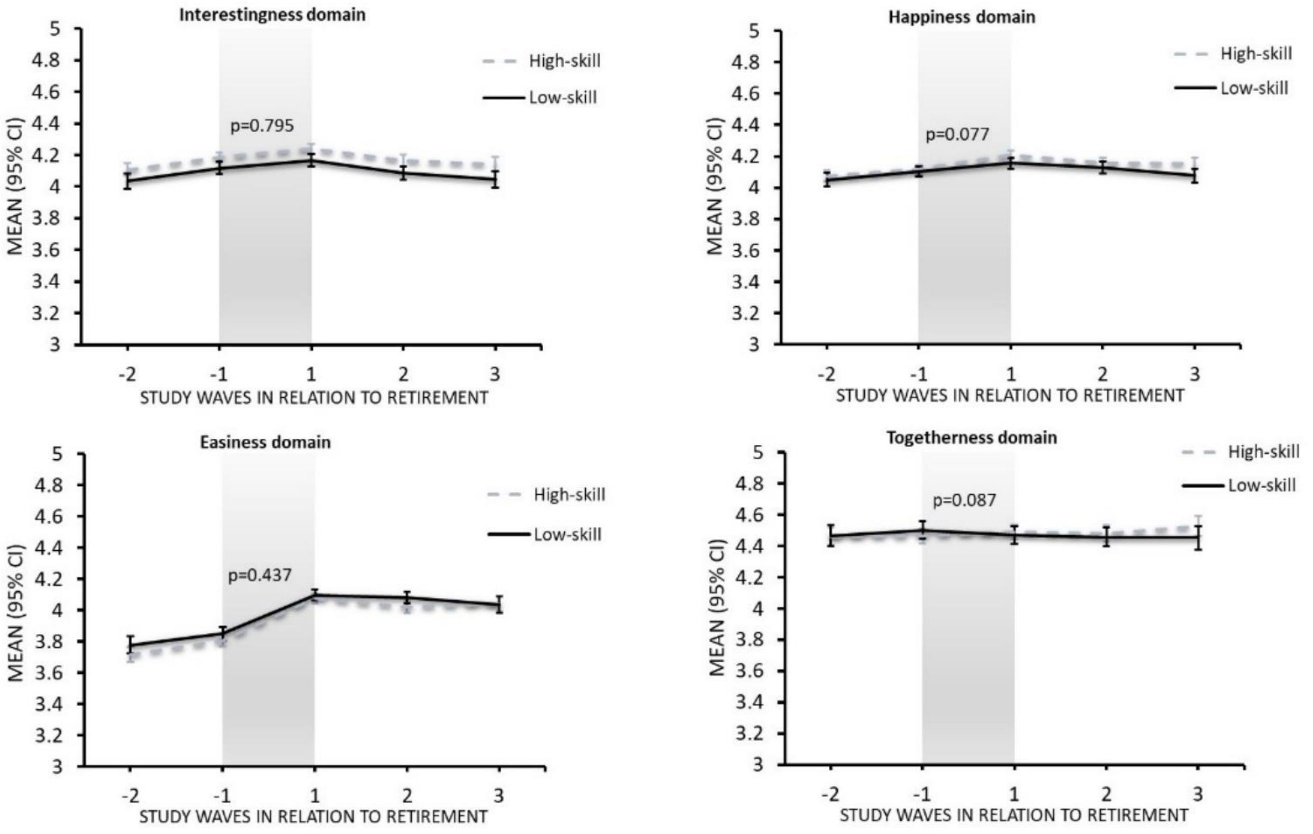

eFigure 2: Mean (95% CI) domain specific life satisfaction scores (score: 1-5) before retirement, during retirement transition and after retirement **based on occupational categories**; p-values for interaction of occupation with time; waves −1 to +1 indicate retirement transition period (study waves are one year apart from each other)

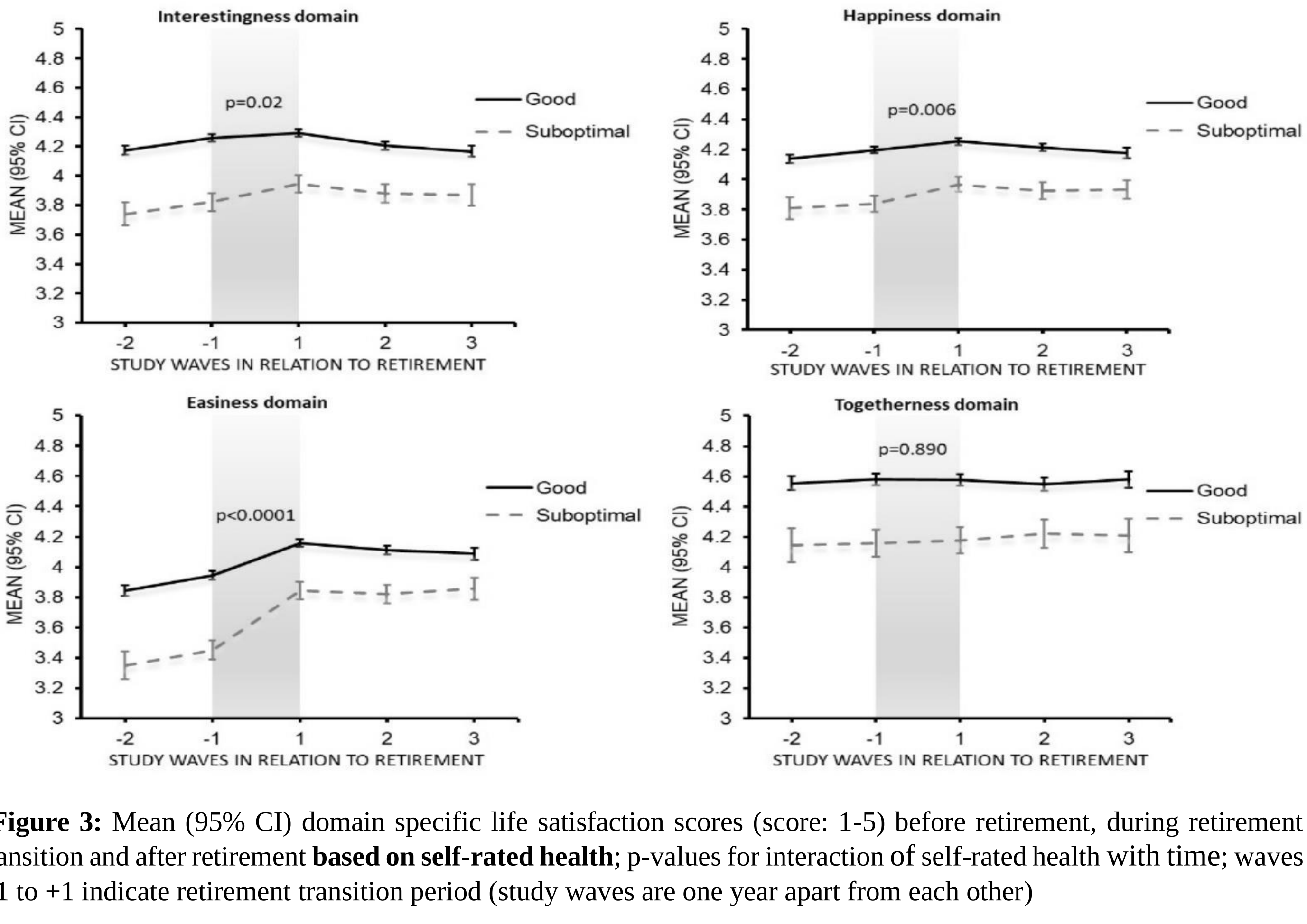

eFigure 3: Mean (95% CI) domain specific life satisfaction scores (score: 1-5) before retirement, during retirement transition and after retirement **based on self-rated health**; p-values for interaction of self-rated health with time; waves −1 to +1 indicate retirement transition period (study waves are one year apart from each other)

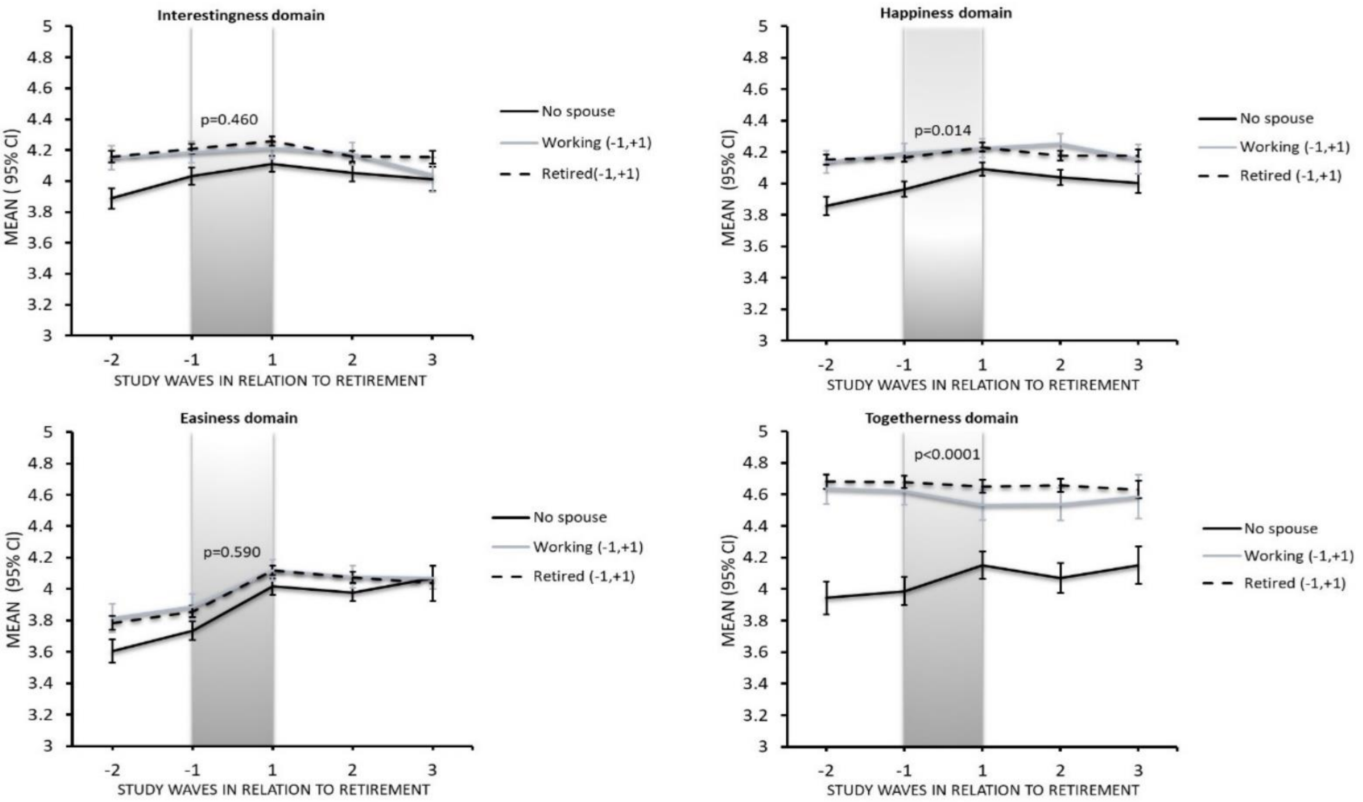

eFigure 4: Mean (95% CI) domain specific life satisfaction scores (score: 1-5) before retirement, during retirement transition and after retirement **based on spousal working status**; p-values for interaction of spousal work status with time; waves −1 to +1 indicate retirement transition period; *spousal working status*: "*No spouse*: never married, divorced or separated and widowed", "*Working (−1,+1)*: spouse working full time at both time points wave −1 and wave +1", ", and "*Retired (−1, +1)*: spouse retired at both time points wave −1 and wave +1" (study waves are one year apart from each other)

eTable3: Domain specific life satisfaction scores (mean estimate and 95% CI) before retirement (wave -1) by pre-retirement characteristics of the study population

Pre-retirement characteristics	Mean (95% CI)							
	**Interestingness**[a]	***p*-value**[*]	**Happiness**[a]	***p*-value**[*]	**Easiness**[a]	***p*-value**[*]	**Togetherness**[a]	***p*-value**[*]
**Total**	4.00 (3.96, 4.04)		3.98 (3.94, 4.01)		3.72 (3.68, 3.77)		4.22 (4.16, 4.28)	
**Gender**		0.362		0.823		0.001		0.308
Women	4.02 (3.98, 4.05)		3.98 (3.95, 4.01)		3.66 (3.62, 3.70)		4.25 (4.20, 4.29)	
Men	3.99 (3.92, 4.05)		3.99 (3.93, 4.04)		3.79 (3.72, 3.87)		4.19 (4.10, 4.29)	
**Occupational category**		0.763		0.224		0.0003		0.008
High skill	4.00 (3.96, 4.02)		3.97 (3.93, 4.01)		3.67 (3.62, 3.72)		4.17 (4.10, 4.23)	
Low skill	4.00 (3.95, 4.05)		4.00 (3.95, 4.04)		3.78 (3.73, 3.84)		4.27 (4.20, 4.34)	
**Self-rated health**		<0.0001		<0.0001		<0.0001		<0.0001
Good	4.21 (4.17, 4.25)		4.16 (4.12, 4.19)		3.98 (3.94, 4.03)		4.43 (4.37, 4.49)	
Suboptimal	3.79 (3.73, 3.85)		3.81 (3.76, 3.86)		3.47 (3.41, 3.54)		4.01 (3.93, 4.09)	
**Spousal working status**		<0.0001		<0.0001		0.005		<0.0001
No spouse	3.92 (3.86, 3.98)[b]		3.89 (3.84, 3.93)[b]		3.67 (3.61, 3.74)[b]		3.89 (3.81, 3.97)[b]	
Working (−1, +1)	4.05 (3.98, 4.13)[b]		4.08 (4.02, 4.14)[b]		3.78 (3.70, 3.86)[b]		4.49 (4.39, 4.60)[b]	
Retired(−1, +1)	4.09 (4.05, 4.13)[b]		4.08 (4.04, 4.12)[b]		3.78 (3.73, 3.83)[b]		4.57 (4.50, 4.63)[b]	

Note: CI, Confidence Interval; −1, year before retirement & +1, year after retirement; [a] models adjusted for age, gender, occupation, self-rated health and marital status; [b]models adjusted for age, gender, occupation and self-rated health; [*]p-values for group difference; *spousal working status*: "*No spouse*: never married, divorced or separated and widowed", "*Working (−1,+1)*: spouse working full time at both time points wave −1 and wave +1", and "*Retired (−1, +1)*: spouse retired at both time points wave −1 and wave +1"
